# Mesoporous Silica Gel–Based Mixed Matrix Membranes for Improving Mass Transfer in Forward Osmosis: Effect of Pore Size of Filler

**DOI:** 10.1038/srep16808

**Published:** 2015-11-23

**Authors:** Jian-Yuan Lee, Yining Wang, Chuyang Y. Tang, Fengwei Huo

**Affiliations:** 1 Key Laboratory of Flexible Electronics (KLOFE) & Institute of Advanced Materials (IAM), Jiangsu National Synergistic Innovation Center for Advanced Materials (SICAM), Institute of Advanced Materials, Nanjing Tech University (NanjingTech), Nanjing 211816, P. R. China; 2 Nanyang Environment & Water Research Institute, Interdisciplinary Graduate School, Nanyang Technological University, Singapore, 639798; 3 Singapore Membrane Technology Centre, Nanyang Environment & Water Research Institute, Nanyang Technological University, Singapore, 637141; 4 Department of Civil Engineering, the University of Hong Kong, Pokfulam, Hong Kong.

## Abstract

The efficiency of forward osmosis (FO) process is generally limited by the internal concentration polarization (ICP) of solutes inside its porous substrate. In this study, mesoporous silica gel (SG) with nominal pore size ranging from 4–30 nm was used as fillers to prepare SG-based mixed matrix substrates. The resulting mixed matrix membranes had significantly reduced structural parameter and enhanced membrane water permeability as a result of the improved surface porosity of the substrates. An optimal filler pore size of ~9 nm was observed. This is in direct contrast to the case of thin film nanocomposite membranes, where microporous nanoparticle fillers are loaded to the membrane rejection layer and are designed in such a way that these fillers are able to retain solutes while allowing water to permeate through them. In the current study, the mesoporous fillers are designed as channels to both water and solute molecules. FO performance was enhanced at increasing filler pore size up to 9 nm due to the lower hydraulic resistance of the fillers. Nevertheless, further increasing filler pore size to 30 nm was accompanied with reduced FO efficiency, which can be attributed to the intrusion of polymer dope into the filler pores.

Forward osmosis (FO) is one of the promising membrane filtration technologies for potable water production^1^,^2^, wastewater treatment and membrane bioreactor (MBR)^3^,^4^,^5^,^6^,^7^,^8^,^9^,^10^,^11^, seawater/brackish water desalination^12^,^13^,^14^, drug delivery^15^, food/juice processing^16^,^17^, and valuable materials enrichment^17^,^18^,^19^,^20^. In FO processes, an osmotic pressure difference is created between a low concentration solution and a high concentration solution to draw water through a semi-permeable FO membrane. Osmotically-driven FO membrane processes can potentially reduce the energy consumption of current technologies^1^,^2^ compared to high pressure-driven reverse osmosis (RO) and nanofiltration (NF) membrane processes. Based on previous studies, several additional advantages of FO processes has been reported, including more reversible fouling due to the no requirement of pressure, high rejection of various solutes and contaminants, and the lack of high temperature or pressure that can potential destroy some sensitive feed stock in certain applications.

Several reviews on FO pointed to the critical need for fabricating high performance FO membranes^1^,^2^,^21^. One commonly adopted approach to enhance FO performance is to create rejection layers with higher water permeability and/or lower salt permeability. Existing attempts include interfacial polymerization (IP) optimization^22^,^23^,^24^,^25^, membrane post-modification by chlorination^26^, and non-IP based approaches, such as layer by layer (LbL)^27^,^28^,^29^ and integral asymmetric FO membranes^30^,^31^,^32^. Another approach is based on nanoparticle incorporation^33^,^34^,^35^,^36^. Inspired by the great success of nanocomposite membranes for reverse osmosis applications^37^,^38^, Ma *et al.* developed microporous zeolite loaded thin film nanocomposite (TFN) FO membranes and demonstrated their significantly higher water permeability and thus FO water flux as a result of different zeolite loading^39^. In a typical design, the pore size of the nanoparticles is chosen to be smaller than the hydrated radius of target solutes to maintain or even enhance solute rejection^37^,^38^.

In parallel to the development of TFN FO membranes, recent studies have shown that the loading of porous fillers to FO substrates are highly effective in enhancing FO performance^40^,^41^,^42^. These porous particles in these mixed matrix substrate (MMS) are shown to provide preferential water paths, which effectively enhances the mass transfer in the membrane support layer and mitigates internal concentration polarization (ICP)^40^. The pore size of the fillers plays a critical role on their effectiveness in enhancing FO performance. Unlike nanoparticle fillers for TFN rejection layers where smaller particle pore size is favored due to solute rejection considerations^37^,^38^, fillers for MMS with large pores are presumably more effective “water channels” due to their much reduced hydraulic resistance. Unfortunately, there is lack of guiding principle for the selection of pore size of filler in the FO substrate.

Therefore, the objective of current study is to systematically study the effects of pore size of substrate fillers on the physiochemical properties, intrinsic separation properties, and FO performance of mixed matrix FO membranes. More specifically, silica gel (SG) particles with a variety of pore size were incorporated into polyacrylonitrile (PAN) to prepare the SG-based mixed matrix membranes (MMMs) due to their hydrophilic nature and a variety of pore size are available. To the best knowledge of the authors, this is the first study to systematically investigate the effects of filler pore size on SG-based mixed matrix FO membranes.

## Results

The nitrogen adsorption and desorption isotherms of different pore size of silica gel is shown in Fig. S1 of the Supporting Information. The measured pore size and BET area are presented in Table 1. For the different pore size of silica gel, the nominal pore size ranged from SG _F40_ (40 Å) to SG _F300_ (300 Å). The measured pore size agreed reasonably well with the nominal pore size. Larger pore size of silica gel was generally associated with smaller BET surface area. For example, SG _F300_ had the largest nominal pore size (300 Å) but also the smallest BET surface area (68 m^2^/g).

The properties of SG-based mixed matrix FO membranes with different pore size are shown in Table 2. The membrane thickness was not significantly influenced by the pore size of SG particles. The average thickness of SG-based mixed matrix membrane (42–49 μm) was similar to the thickness of commercial available HTI cellulose acetate FO membranes (50 μm) but was much thinner than HTI TFC FO membranes (100 μm) and traditional TFC RO membranes (150 μm)^25^,^43^. In all cases, the incorporation of SG particles had positive effect in improving the hydrophilicity of the substrate. The contact angle of SG-loaded substrates ranged from 33–47°, as compared to 54° for the control without SG addition. The SG-based mixed matrix FO membranes were also more hydrophilic compared to the commercial HTI CA FO membranes (contact angle ~62°). The beneficial effect of nanoparticle loading on hydrophilicity enhancement is consistent with prior studies^39^,^44^, and it is expected the increased substrate hydrophilicity could enhance FO water flux.

**Figure 1:**
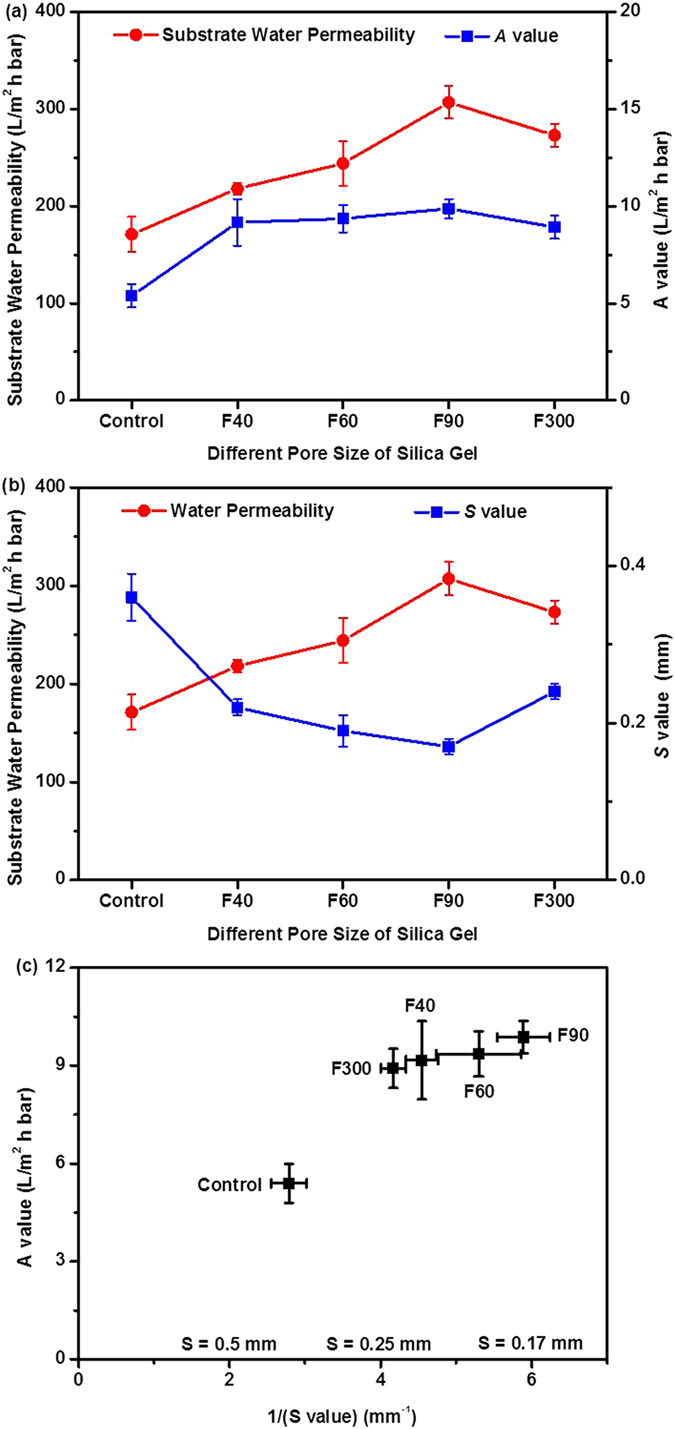
The effect of different pore size of SG-based mixed matrix FO membranes on (a) substrate water permeability and *A* value; (b) substrate water permeability and *S* value; and (c) the plot of *A* value against the 1/*S* value. Testing conditions: DI water as the feed solution; applied pressure: 10 bars, error bar was based on the standard deviation of 3 replicate measurements.

Adding SG particles to the substrate had no obvious effect on the membrane bulk porosity (Table 2). Nevertheless, the substrate pure water permeability (PWP) was substantially increased for the SG-loaded substrates. For the substrate F90, its PWP was nearly double of that of the control. Since both substrates had similar thickness and bulk porosity, the enhanced PWP was most likely attributed to increased surface porosity (*ε*_*s*_) and/or reduced pore tortuosity (*τ*). According to the classical Hagen-Poiseuille equation, the substrate PWP is proportional to the ratio of *ε*_*s*_/*τ*. The water permeability of the resulting LbL membranes (*A* value) followed a similar trend to that of the substrate PWP (Fig. 1a), suggesting an important dependence of the rejection layer properties on the substrate pore structure. This observation is consistent with previous studies^40^,^45^, which can be partially attributed to the reduced effective water transport distance through the rejection layer for a substrate with higher PWP. The solute (MgCl_2_) rejection decreased as the membrane permeability increased, showing a typical permeability-selectivity tradeoff^46^,^47^. This observation suggests that salt rejection of a mixed matrix membrane is sensitive to the pore size of the SG particles even though these particles are only present in the membrane substrate and not in the rejection layer. Therefore, additional research studies are needed to further investigate the mechanisms involved and further study is needed to optimize the salt rejection of SG-based of mixed matrix FO membrane without compromising the water permeability.

It is also interesting to note the substantially reduced structural parameter (*S*) for the SG-based mixed matrix FO substrates (Table 2). Structural parameter is a critical FO membrane property, with a lower *S* value preferred for controlling ICP in the FO substrate. In the current study, the *S* value was reduced from 0.36 mm (control) to 0.17–0.24 mm (MMMs) as a result of SG loading. In all cases, the SG-based mixed matrix FO substrates had much lower *S* values compared to the commercial HTI FO membranes (0.47 mm for CTA membrane and 0.62 mm for TFC membrane)^25^,^43^. While their *S* value was similar to that of the-state-of-the-art FO membranes supported by highly porous nanofiber substrates^48^,^49^, MMMs generally offer much better mechanical stability^45^,^50^ that can potentially allow these membranes to be used for more demanding applications such as pressure retarded osmosis^51^,^52^ and pressure assisted osmosis^53^.

The effect of filler pore size (*d*_*filler*_) on *S* value is shown in Fig. 1b. Increasing *d*_*filler*_ up to 90 Å (F90) helped to reduce the *S* value, which is consistent with the hypothesis that the mesoporous silica gels with larger *d*_*filler*_ are more effective in enhancing mass transfer in the substrate since they are more permeably to water and solutes. Nevertheless, the *S* value increased from 0.17 ± 0.01 mm to 0.24 ± 0.01 mm when *d*_*filler*_ was further increased from 90 Å (F90) to 300 Å (F300). One plausible explanation is that, at very large filler pore size, the polymer molecules can enter into the filler pores to result in partial pore blockage and therefore reduced effectiveness of the fillers. Figure S2 shows the nitrogen adsorption and desorption isotherms of clean F300 silica gels and that of F300 fillers separated from a polymer dope solution by centrifugation. The results clearly indicate a significant reduction of both the filler pore size from 17 nm to 15 nm and BET surface area from 68 m^2^/g to 54 m^2^/g, which providing a direct support to the filler pore blockage hypothesis. The current study seems to suggest that the optimal *d*_*filler*_ value is dependent on the competing effects of the reduced resistance to water/solute transport for larger pore size and the simultaneously increased risk of blockage of filler pores by the polymer chains. *Based on the observation of the current study, an optimal filler pore size of ~9 nm is recommended.* It is worthwhile to note that this optimal value may depend on the properties of the polymer dope and the filler particles. For example, classical theory predicts that the liquid (or in this case the polymer dope) entry pressure into a pore is given by 4*γ*cos*θ/d*_*filler*_, where *γ* is surface tension of the polymer dope and *θ* is the contact angle. Thus, a more viscous polymer dope with higher surface tension may lead to a higher optimal *d*_*filler*_ value. Further systematic studies are required on understanding the effect of polymer dope properties on the optimal filler pore size.

Both substrate PWP and *S* values are presented in Fig. 1b as a function of filler pore size. Clearly, PWP and *S* value were strongly correlated. The PWP curve and *S* value curve were nearly mirror images to each other. As discussed earlier in the section, the substrate PWP is proportional to the ratio of *ε*_*s*_/*τ*. Meanwhile, based on classical ICP theory^54^,^55^, *S* value is proportional to tortuosity, and it is inversely proportional to the substrate porosity. Recent research further reveals that the *S* value is generally dominated by the skin layer contribution (as a result of much lower surface porosity compared to the average bulk porosity)^32^,^40^,^56^. Thus, it is reasonable to expect a strong dependence of *S* value on *τ*/*ε*_*s*_, which explains the inverse relationship between substrate PWP and *S* value. Figure 1c plots the water permeability (*A* value) of SG-based mixed matrix FO membranes as a function of 1/*S* value. *A* value and *S* value are two key parameters dictating the FO water flux. A linear relationship between the *A* value and 1/*S* was observed. The current study clearly demonstrated that the feasibility of using mixed matrix substrates to simultaneously increase *A* value and reduce *S* value by enhancing the substrate mass transfer.

**Figure 2:**
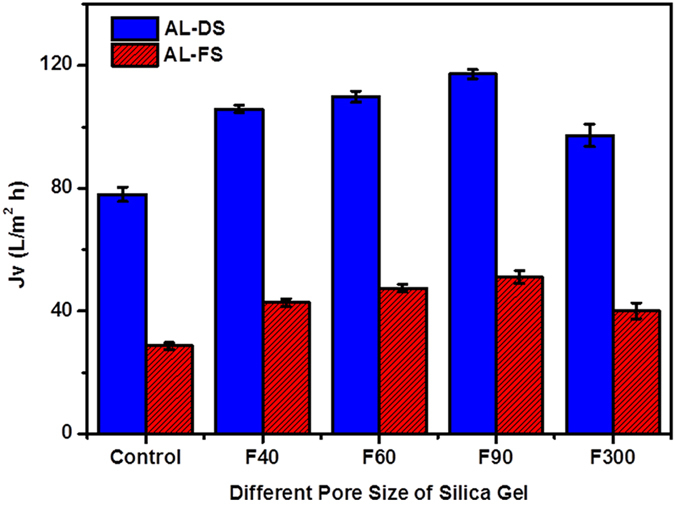
The effect of different pore size and of silica gel loading on FO water flux of SG-based mixed matrix FO membranes. Testing conditions: 0.5 M MgCl_2_ as the draw solution and DI water as the feed solution, error bar was based on the standard deviation of 3 replicate measurements.

Figure 2 demonstrates the effect of different pore size of SG on FO water flux and the *Js/Jv* ratio of SG-based mixed matrix FO membranes is shown in Fig. S3 of the Supporting Information. The FO water flux was tested for both AL-DS and AL-FS orientations using 0.5 M MgCl_2_ as DS and DI water as FS. Without the presence of SG particle in the substrate, the control membrane had an FO water flux of 78.1 L/m^2^ h in AL-DS. A lower flux of 28.7 L/m^2^ h was obtained in AL-FS due to the more severe dilutive ICP in this orientation^1^. For both AL-DS and AL-FS orientations, the FO water flux of the control membrane was significantly lower than those values of the SG-based MMM membranes, suggesting that the incorporation of SG particles is an effective approach for enhanced FO water flux performance. The optimal FO water flux was obtained for the filler pore size of 90 Å, and further increasing *d*_*filler*_ to 300 Å caused an FO water flux reduction. As explained in the previous section, the pores of SG particle could be clogged with polymer due to the large pore of SG particle, which lead to less effective water transport inside the substrate.

The FO water flux of SG-based mixed matrix FO membranes (F90) is presented in Fig. S4 for a variety of DS concentrations (0.1, 0.3, 0.5, 1.0, 3.0 M MgCl_2_) and FS concentrations (0, 10, 100 mM NaCl). From the viewpoint of driving force, higher the concentration of the DS should result in higher the FO water flux for the SG-based mixed matrix FO membranes. This is because of the larger osmotic pressure was created across the membrane regardless of the membrane orientation. In AL-DS orientation, the FO water flux of SG-based mixed matrix FO membranes (F90) was 42.3 L/m^2^ h for a 0.1 M MgCl_2_ and it increased to 117.2 L/m^2^ h at 0.5 M MgCl_2_ as a result of increased the osmotic driving force. The highest FO water flux of 126 L/m^2^ h was achieved by using the 1.0 M MgCl_2_ as the DS and DI water as the FS, this clearly demonstrates the potential of SG-based mixed matrix FO membranes for high flux FO application. However, further increase the concentration of DS to 3.0 M MgCl_2_ did not result in additional gain in FO water flux. This is consistent with prior studies^14^,^57^ that ICP increases and FO efficiency decreases at higher DS concentrations. The membrane also worked well at higher FS concentration (82.5 L/m^2^ h and 67.4 L/m^2^ h in AL-DS orientation and 46.2 L/m^2^ h to 41.2 L/m^2^ h in AL-FS orientation using 10 mM or 100 mM NaCl solution as FS and 1.0 M MgCl_2_ as DS).

## Discussion

**Figure 3:**
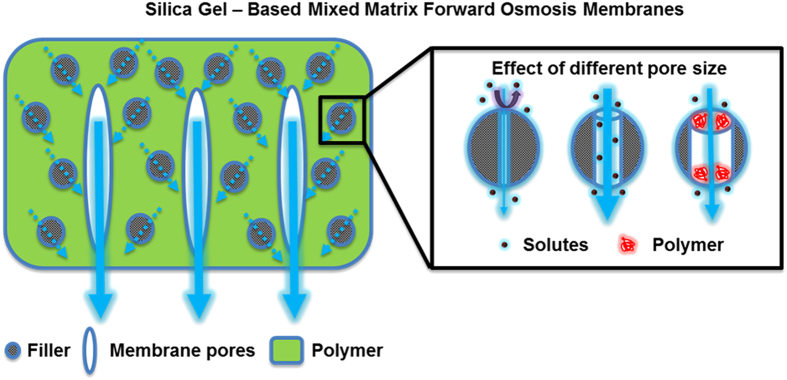
Proposed mechanism for the enhanced FO water flux in SG-based mixed matrix FO membranes.

SG-based mixed matrix FO membranes with different pore size of SG particles were systematically fabricated and characterized in this current study. The pore size of SG particles played an important role in the current study. For SG particles with larger pore size (up to ~9 nm), the corresponding membrane had higher water permeability as well as lower structural parameter than the SG particles with smaller pore size. This clearly indicates the importance of water transport through the internal pores of the SG particles, which provide additional pathways (i.e., shortcuts) for water molecules to pass through the support layer (Fig. 3). While earlier works have focused on the use of microporous fillers (*d*_*filler*_ < 2 nm)^40^, the current study clearly demonstrates the beneficial effect of larger *d*_*filler*_. Unlike the case of TFN membranes where the fillers are designed in such a way that they retain solutes while allowing water to permeate through them, larger *d*_*filler*_ is preferred for fillers to the membrane substrate as the latter is designed to be conduits to both water and solute molecules. Nevertheless, caution should be exercised to avoid overly large filler pores to prevent their intrusion by polymer dope. The current study shows that a simultaneous improvement of membrane water permeability and reduction in structural parameter can be realized by the porous filler based MMM approach, thereby providing an additional degree of freedom to FO performance enhancement. A similar strategy might be used for the fabrication of SG-based mixed matrix pressure-retarded osmosis (PRO) membranes to control the ICP effect in the PRO substrate. Compared to FO, PRO has great requirement of membrane mechanical stability^51^,^52^. In this regard, the MMM approach is more promising approach (say compared to nanofiber based approach) for preparing membranes to greater mechanical strength. Existing works showed that mixed matrix strategy could significantly enhance not only the tensile strength but also the toughness of the membrane for a variety of applications, such as pervaporation and direct methanol fuel cells^58^,^59^.

## Methods

### Materials

Unless otherwise stated, all chemicals and reagents were used as received. Polyacrylonitrile (PAN, Sigma–Aldrich, Mw ~ 150,000, Lot# MKBD6325V) was used for polymer solution preparation due to its ease of processing, good chemical resistance, mechanical strength, and thermal stability PAN in the current study^27^,^60^. *N*,*N*-dimethylformamide (DMF, purity ≥ 99.8%, Merck) was used as solvent to dissolve PAN and lithium chloride (anhydrous LiCl, Merck) was added to promote the formation of finger-like pores^42^. In order to increase the surface hydrophilicity and surface charge, sodium hydroxide (anhydrous NaOH, purity ≥ 98%, Merck) was used to prepare alkali solution for base treatment of PAN substrate. Silica gel (SiliCycle Inc., Canada) were used for MMMs preparation due to its high thermal and chemical stability, high surface area, neutral pH, non-toxic, tunable particle and pore size, readily available in kilogram scale, commercial viable and well characterized properties^61^,^62^. According to the product characteristics provided by the company, the pore size of silica gel ranged from 40 to 300 Å respectively (Table 1).

For the layer-by-layer (LbL) self-assembly, poly(allylamine hydrochloride) (PAH, *Mw*  ∼ 112,000–200,000, Polyscience, Lot# 639458) was used as positively charged polyelectrolyte and poly(sodium 4-styrene-sulfonate) (PSS, *Mw* ~ 70,000, 30 wt.% in H_2_O, Sigma–Aldrich, Batch# 09622HH) was used as negatively charged polyelectrolyte for the formation of rejection layer. The ionic strength of the polyelectrolyte solution was controlled by adding sodium chloride (NaCl, 99%, Merck). Glutaraldehyde (GA, 25% in water, Sigma–Aldrich) is used as a crosslinking reagent to make a salt rejection layers with crosslinked layer-by-layer (xLbL) film, which have relatively high FO water flux and good rejection to divalent ions and may have potential applications in biomass concentration, food processing, etc^27^,^28^,^29^,^63^.

### Fabrication of SG-based Mixed Matrix FO Substrates

The detailed procedure for the fabrication of SG-based mixed matrix FO substrates can be found elsewhere^42^. First of all, silica gel particles (1.0 wt.% based on the total weight of dope solution) were added into DMF followed by ultrasonicating the solution for 1 h to prevent the aggregation of SG particles in the DMF. Next, adding LiCl (2 wt.%) into the mixture prior to the addition of PAN (18 wt.%). The mixture was stirred at 60 °C for 24 h to obtain the homogenous solution, which was left at room temperature for 12 h to release gas bubbles trapped inside the solution. A thin polymer film was spread out uniformly onto a clean glass plate followed by phase inversion in the tap water bath at room temperature. The resulting substrates were named as Control, F40, F60, F90, and F300 based on the nominal pore size of silica gel (refer to Table 1). In order to partially hydrolyze the surface of SG-based mixed matrix FO membrane substrate, the membrane substrates were immersed into the 1.5 M NaOH solution at room temperature for 1.5 h to before the processes of LbL self-assembly and crosslinking.

### Preparation of Rejection Layer of SG-based Mixed Matrix FO Membranes

The preparation of LbL rejection layer can be found in details elsewhere^27^,^28^,^63^,^64^. In brief, one side of the SG-based mixed matrix FO membrane substrates were alternatively immersed into the PAH and PSS solution for 15 min followed by soaking the substrate into the DI water after each polyelectrolyte soaking step to remove the excess charged polyelectrolyte. In order to achieve membranes rejection layer with reasonable salt rejection and high water permeability for FO applications, the PAH/PSS treatment was repeated three times based on the optimal condition of previous studies^27^,^28^,^42^. SG-based mixed matrix FO membranes were then cross-linked by immersing the membranes in a 0.1 wt.% GA solution for 30 min followed by soaking with DI water for 5 min to remove excess GA.

### Characterization of SG particle and SG-based Mixed Matrix FO Membranes

The detailed procedure for the characterization of SG particle and SG-based mixed matrix FO membranes can be found in details elsewhere^42^. Briefly, the surface roughness of the SG-based mixed matrix FO membranes was characterized by atomic force microscope (AFM, Park Systems XE-100, Korea)^42^,^65^. The Brunauer-Emmett-Teller (BET) surface area and the pore size of SG particles were characterized by Micromeritics ASAP 2020 instrument (USA). The membrane porosity (*ε*) were measured using gravimetric measurementand the surface hydrophilicity were tested by OCA contact angle goniometer system (DataPhysics Instruments GmbH, Germany)^25^,^66^.

### Performance Evaluation of SG-based Mixed Matrix FO Membranes

The pressurized crossflow system used for determining membrane water permeability and salt rejection has been reported in the previous studies^25^,^39^,^55^. Briefly, the water permeability of SG-based mixed matrix membranes was determined by weighing the amount of permeates at the fixed interval. The conductivity measurements (Ultra Meter II^TM^ 4P, Myron L Company, CA) was used to determine salt rejection by measuring the conductivity difference between the feed water and permeate water. For the evaluation of FO performance, a small piece of membrane with the membrane area of 60 cm^2^ was tested for both the active-layer-facing-DS (AL-DS) and active-layer-facing-FS (AL-FS) orientations. Concentrated MgCl_2_ solutions (0.1, 0.3, 0.5, 1.0, or 3.0 M) were used as draw solution and the DI water, 10 mM or 100 mM NaCl solution was used as feed solution. To determine the FO water flux, the weight change of the FS tank was measured with a weighing machine at fixed time intervals. On the other hand, the FO solute flux was determined by measuring the conductivity of the feed solution. Finally, the structural parameter *S* of the FO membranes was determined by using the equations in the previous study:^27^


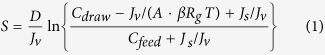



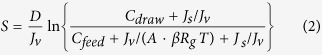


where *D* is the solute diffusion coefficient; *S* is the structural parameter of the support layer; *C*_*draw*_ and *C*_*feed*_ are the concentrations of draw solution and feed solution, *J*_*s*_ is the solute flux; 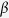 is the van’t Hoff coefficient; *R*_*g*_ is the universal gas constant; *T* is the absolute temperature. In the current study, the equation of AL-FS orientation was used to calculate the *S* value.

## Additional Information

**How to cite this article**: Lee, J.-Y. *et al.* Mesoporous Silica Gel–Based Mixed Matrix Membranes for Improving Mass Transfer in Forward Osmosis: Effect of Pore Size of Filler. *Sci. Rep.* **5**, 16808; doi: 10.1038/srep16808 (2015).

## Supplementary Material

Supporting Information

## Figures and Tables

**Table 1:** The properties of different pore size and BET surface area of silica gel filler.

SG^a^	Nominal Pore Size (Å)	Measured Pore Size (Å)	BET Surface Area (m^2^/g)
SG _F40_	40	58	433
SG _F60_	60	59	424
SG _F90_	90	106	318
SG _F300_	300	173	68

^a^All the SG particles have comparable particle sizes, with each ranging from 40–63 μm.

**Table 2:** Membrane thickness, contact angle, membrane bulk porosity, substrate water permeability, membrane water permeability salt rejection, and structural parameter of SG-based MMMs with different pore size of silica gel particles^a^.

Membrane	Membrane Thickness (μm)	Contact Angle (^ο^)	Membrane Bulk Porosity (%)	Substrate Water Permeability^b^ (L/m^2^ h bar)	Membrane Water Permeability^b^ (L/m^2^ h bar)	Salt Rejection^c^ (%)	Structural Parameter (mm)
Control	42 ± 2	54 ± 4	80 ± 2	171 ± 18	5.4 ± 0.6	83 ± 3	0.36 ± 0.03
F40	43 ± 1	47 ± 3	78 ± 2	218 ± 6	9.2 ± 1.2	80 ± 3	0.22 ± 0.01
F60	46 ± 1	39 ± 2	77 ± 4	244 ± 23	9.4 ± 0.7	74 ± 6	0.19 ± 0.02
F90	43 ± 1	33 ± 3	78 ± 3	307 ± 17	9.9 ± 0.5	68 ± 2	0.17 ± 0.01
F300	49 ± 1	42 ± 4	79 ± 2	273 ± 12	8.9 ± 0.6	76 ± 4	0.24 ± 0.01

^a^The experiment errors are reported as the standard deviation of at least 3 repeated measurements.

^b^Measured at an applied pressure of 5 bar with ultrapure water as feed water in the pressurized cross flow filtration testing mode.

^c^Measured at an applied pressure of 5 bar with 5 mM MgCl_2_ as feed water in the pressurized cross flow filtration testing mode.
