# Supplementary material for: Mesoporous Silica Gel–Based Mixed Matrix Membranes for Improving Mass Transfer in Forward Osmosis: Effect of Pore Size of Filler
Source: Sci Rep. 2015 Nov 23;5:16808. doi: 10.1038/srep16808 (PMC4655366; doi:10.1038/srep16808)
Supplement: Supporting Information [file srep16808-s1.pdf]

Cover Page for Supporting Information

Author list:
Jian-Yuan Lee, Yining Wang, Fengwei Huo*, Chuyang Y. Tang*

Manuscript title:
Mesoporous Silica Gel–Based Mixed Matrix Membranes for Improving Mass Transfer in Forward Osmosis: Effect of Pore Size of Filler

Total number of pages (including the cover page):
6 pages

List of Figures:

Page 2 **S1. Nitrogen sorption isotherm of different pore size of silica gel.**

Page 3 **S2. Nitrogen adsorption and desorption isotherm of silica gel F300 before and after pore-filled by polymer.**

Page 4 **S3. The effect of different pore size of silica gel on the ratio of salt flux over water flux of SG-based mixed matrix FO membranes.**

Page 5 **S4. The effect of different concentration of DS concentration; and FS concentration of SG-based mixed matrix FO membranes.**

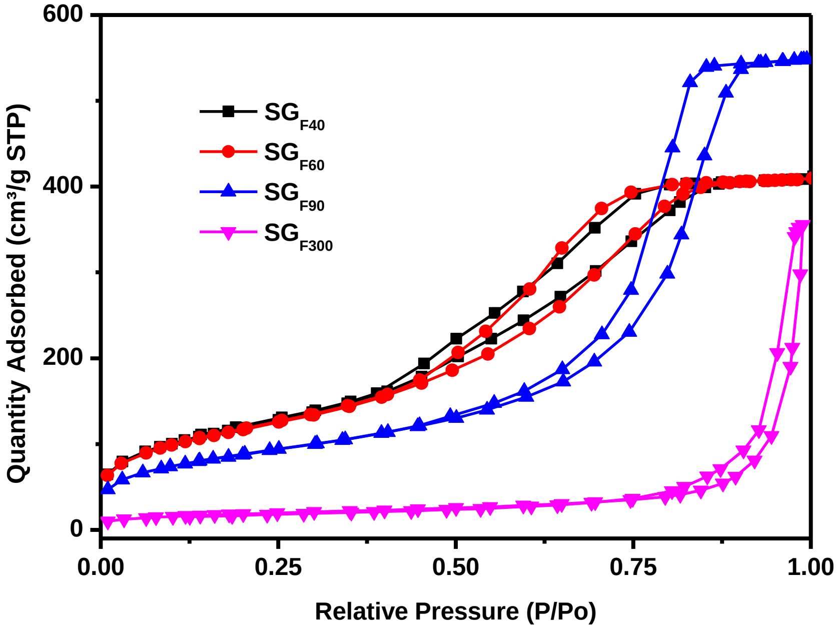

S1. Nitrogen sorption isotherm of different pore size of silica gel.

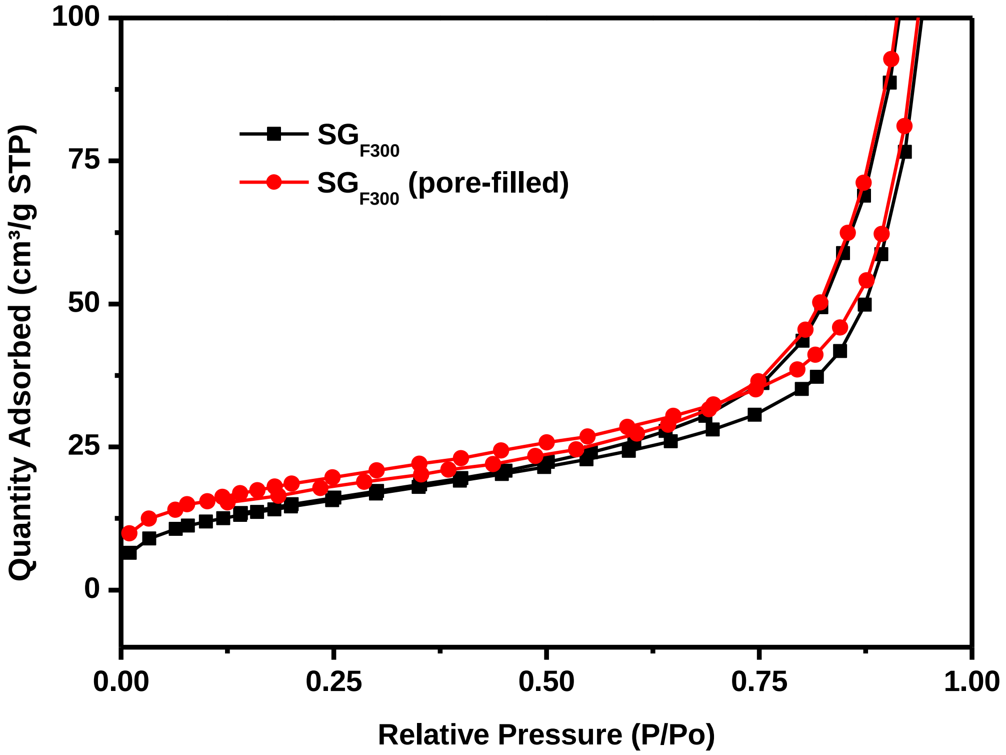

S2. Nitrogen adsorption and desorption isotherm of silica gel F300 before and after pore-filled by polymer.

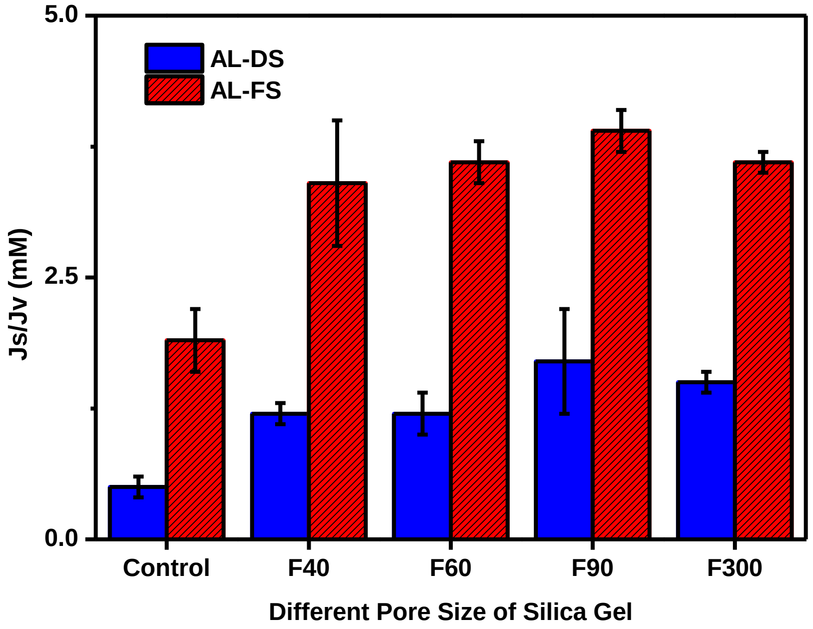

S3. The effect of different pore size of silica gel on the ratio of salt flux over water flux of SG-based mixed matrix FO membranes. Testing conditions: 0.5 M $MgCl_2$ as the draw solution and DI water as the feed solution, error bar was based on the standard deviation of 3 replicate measurements.

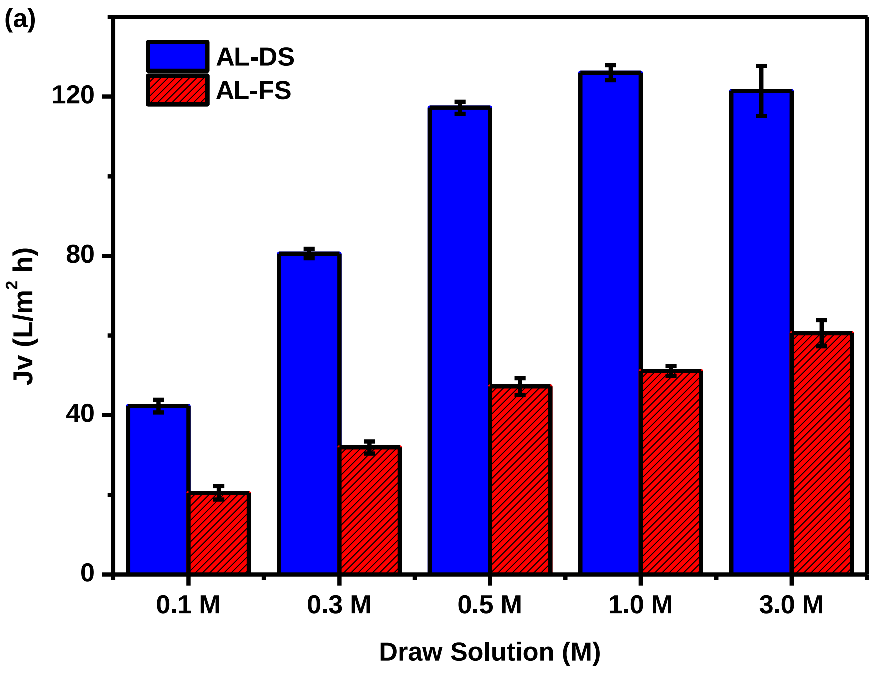
(a)
AL-DS
AL-FS
0
40
80
120
Jv (L/m2 h)
0.1 M
0.3 M
0.5 M
1.0 M
3.0 M
Draw Solution (M)

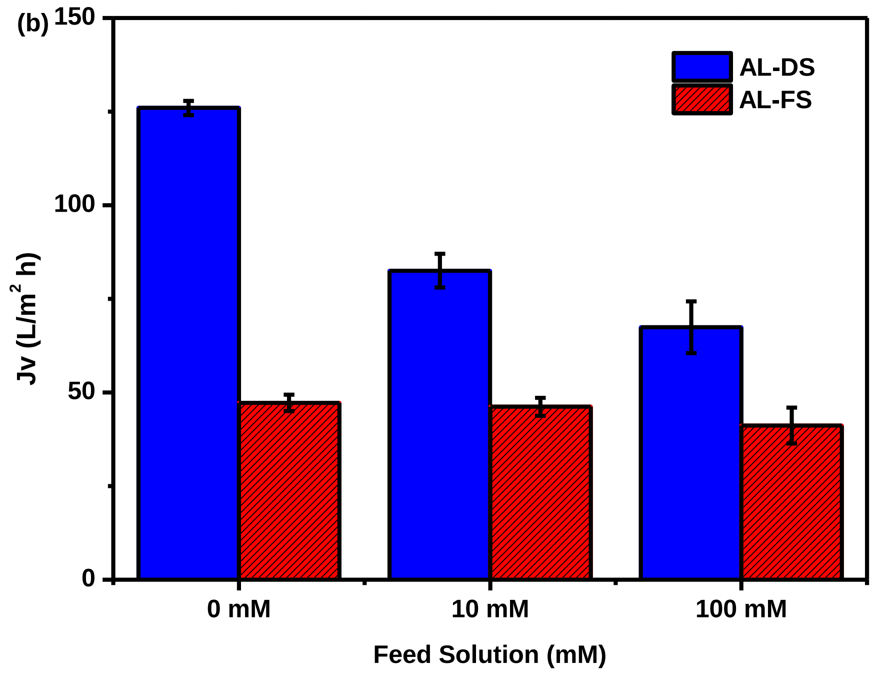
(b)
AL-DS
AL-FS
0
50
100
150
Jv (L/m2 h)
0 mM
10 mM
100 mM
Feed Solution (mM)

S4. The effect of different concentration of (a) DS concentration; and (b) FS concentration of SG-based mixed matrix FO membranes (F90). Testing conditions: (a) concentration of draw solution (0.1 – 3.0 M $MgCl_2$) using DI water as FS; (b) concentration of feed solution (0 - 100 mM NaCl) using 1 M $MgCl_2$ as DS.
